# Examining the effects of a high-protein total diet replacement on energy metabolism, metabolic blood markers, and appetite sensations in healthy adults: protocol for two complementary, randomized, controlled, crossover trials

**DOI:** 10.1186/s13063-019-3950-y

**Published:** 2019-12-27

**Authors:** Camila L. P. Oliveira, Normand G. Boulé, Arya M. Sharma, Sarah Elliott, Mario Siervo, Sunita Ghosh, Aloys Berg, Carla M. Prado

**Affiliations:** 1 Human Nutrition Research Unit, Department of Agricultural, Food & Nutritional Science, University of Alberta, Edmonton, AB Canada; 2 Faculty of Kinesiology, Sport & Recreation, University of Alberta, Edmonton, AB Canada; 3 Division of Endocrinology & Metabolism, Department of Medicine, University of Alberta, Edmonton, AB Canada; 4 Human Nutrition Research Unit, Department of Agricultural, Food & Nutritional Science and Alberta Research Centre for Health Evidence, University of Alberta, Edmonton, AB Canada; 5 School of Life Sciences, Division of Physiology, Pharmacology and Neuroscience, University of Nottingham, Nottingham, England, UK; 6 Department of Medical Oncology, University of Alberta, Edmonton, AB Canada; 7 Faculty of Medicine, University of Freiburg, Freiburg, Germany; 8 Department of Agricultural, Food and Nutritional Science - Division of Human Nutrition, University of Alberta, 4–002 Li Ka Shing Centre for Health Research Innovation (Office 2-021E), Edmonton, Alberta T6G 2E1 Canada

**Keywords:** Protein, Total diet replacement, Energy metabolism, Metabolic biomarkers, Appetite, Adults

## Abstract

**Background:**

High-protein diets and total diet replacements are becoming increasingly popular for weight regulation; however, further research is needed to elucidate their impact on the physiology of body weight regulation. The aim of this inpatient metabolic balance study is to compare the impact of a high-protein total diet replacement versus a control diet (North American) on energy expenditure, macronutrient oxidation rates and balances, metabolic blood markers and appetite sensations in healthy adults.

**Methods:**

Two randomized, controlled, cross-over clinical trials conducted separately in men and women will be conducted. In each trial, participants will be allocated to two isocaloric arms: a) Control diet: 55% carbohydrate, 15% protein, and 30% fat; b) High-protein total diet replacement: 35% of carbohydrate, 40% protein, and 25% fat. They will receive the prescribed diets for 32 h while inside the whole-body calorimetry unit. Diets will be designed to ensure participants are in energy balance. The following physiological changes will be compared between groups: energy expenditure, macronutrient oxidation rates and balances, metabolic blood markers, and appetite sensations. Body composition will be assessed at baseline using dual-energy X-ray absorptiometry.

**Discussion:**

This will be the first inpatient metabolic balance study examining the impact of a high-protein total diet replacement on energy metabolism, metabolic blood markers and appetite sensations in healthy young adults (of both sexes) using a whole-body calorimetry unit. Results of this clinical trial can ultimately be used to develop strategies to optimize high-protein diet interventions and weight management.

**Trial registration:**

ClinicalTrials.gov Identifiers: NCT02811276 (registered on 16 June 2016) and NCT03565510 (registered on 11 June 2018).

**Protocol version:**

NCT02811276: version 10 (2 March 2018); NCT03565510: version 3 (28 September 2018).

## Introduction

Defining the ideal macronutrient composition to prevent and treat obesity and its related diseases has been the target of many studies dating back to the 1950s. At that time, Rubner and Atwater discovered that macronutrients (carbohydrate, protein, fat, and alcohol) can elicit different responses in energy expenditure [1]. Later, the energy expended to absorb, process, and store ingested nutrients was determined to be highest for protein (25–30% of energy content), followed by carbohydrate (6–8%), and fat (2–3%) [2], suggesting that a diet with a protein content above the recommended values (i.e., for healthy adults > 19 years of age: 0.80 g/kg of body weight/day or 10 to 35% of the total energy intake) [3] may be advantageous for weight management when compared to other diets with different proportions of macronutrients [4]. Furthermore, protein appears to exert a stronger satiating effect and decrease energy intake under *ad libitum* conditions [5]. Therefore, high-protein (HP) diets are becoming increasingly popular for weight loss; however, lack of dietary adherence due to behavior and/or environmental factors seems to impact long-term effectiveness of this type of diet on weight-loss maintenance [6].

Total diet replacements (TDR) are nutritionally complete formula foods designed to replace the whole diet for a specific period of time to facilitate weight loss. Insufficient evidence on the long-term effectiveness of this approach hinders its recommendation for the treatment of obesity [7]. Astbury et al. [8] showed that participants with obesity following a TDR weight loss program lost 7.2 kg more than individuals receiving usual care after 1 year of intervention and experienced greater improvements in biomarkers of cardiovascular and metabolic risk. In a similar study, Lean et al. [9] observed that half of participants on a 1-year TDR weight management program reverted to a non-diabetic state, and remission was shown to be closely related to the degree of weight loss.

Considering the benefits of a higher protein intake and TDR in isolation, the combination of these strategies might generate synergistic effects, promoting effective weight loss and its maintenance over time. However, to consider a HP TDR as an option to prevent and treat obesity, it is imperative to first understand the physiological impact of this strategy in a healthy population group so that the effects are better translated in individuals with obesity and its related chronic diseases. Of particular importance is the study of these variables separately in men and women, as their metabolic regulation differs considerably [10–12]. Therefore, inpatient controlled-feeding research using state-of-the-art technology, such as the whole-body calorimetry unit (WBCU), is needed to elucidate the exact role of HP diets comprising a TDR on the regulation of energy expenditure, macronutrient oxidation rates and balances, appetite sensations, and metabolic blood markers. To our knowledge, no research of this kind has been conducted separately in healthy men and women. Therefore, the aim of this inpatient metabolic balance study is to compare the impact of an HP-TDR versus a control (CON) diet (North American) on energy expenditure, macronutrient oxidation rates and balances, metabolic blood markers and appetite sensations in male and female healthy adults.

## Methods

### Study design and ethical procedures

These studies will be randomized, controlled, crossover-designed, clinical trials conducted separately in women and men at the Human Nutrition Research Unit (HNRU), University of Alberta (Edmonton, AB, Canada). The corresponding research protocol fulfils the requirements of the Standard Protocol Items: Recommendations for Interventional Trials (SPIRIT) checklist [13] (Additional file 1). The research protocols involving women and men were approved separately by the University of Alberta Ethics Board (Pro00066006 and Pro00083005) and complied with the standards as set out in the Canadian Tri-Council Policy statement on the use of human participants in research. If important protocol modifications are needed, the research team will contact the University of Alberta Ethics Board, trial registry (clinicaltrials.gov), and trial participants.

### Outcome measures

The primary outcome is as follows:

- Difference in fat balance (i.e., fat intake minus fat oxidation) between the HP-TDR and CON conditions

The secondary outcome is as follows:

- Difference in the 24-h energy expenditure (EE) between the HP-TDR and CON conditions

The exploratory outcomes are as follows:

- Difference in selected components of energy metabolism (resting metabolic rate [RMR], basal metabolic rate [BMR], sleeping metabolic rate [SMR], postprandial EE, exercise EE, 24-h respiratory quotient [RQ], macronutrient oxidations rates, and energy and macronutrient balances) between the HP-TDR and CON conditions
- Change in concentrations of metabolic blood markers (glucose, insulin, cholesterol, high-density lipoprotein [HDL] cholesterol, low-density lipoprotein [LDL] cholesterol, triglyceride and non-HDL cholesterol, acyl-ghrelin, leptin, peptide YY [PYY], glucagon-like peptide 1 [GLP-1], free glycerol and non-esterified fatty acids [NEFA]) over time (within-groups) and between the HP-TDR and CON conditions
- Difference in appetite sensations (hunger, satiety, fullness, and prospective food consumption) between the HP-TDR and CON conditions

The primary outcome of this study was initially planned to be the RQ. However, it was changed to fat balance (i.e., fat intake minus fat oxidation) *before* any data analysis. The rational for the change was that RQ had been established to be strongly associated to the dietary carbohydrate content [14], and the fat balance was thought to be a more meaningful outcome.

### Data management

Before participating in the study, all participants will be informed of the procedures and potential risks involved in the investigation and will be asked to provide written informed consent. Moreover, participants will be informed that all data collected during the study will be kept private, and no personal identifiable information will be shared. All data will be securely stored for 5 years. Storage of research-related paper files will be in a locked cabinet at the HNRU, and all electronic files will be stored on a secured server. If participants decide to discontinue the study, no other information will be collected; however, the data already collected will be kept, unless participants specifically request that it be destroyed. Only the study team will have access to all hard copy and electronic datasets.

### Research participants

Healthy women and men aged 18 to 35 years and with a body mass index (BMI) between 18.5 and 24.9 kg/m^2^ will be recruited via advertisements placed on notice boards at the University of Alberta north campus. All participants will be nonsmokers, and women will be required to have a regular menstrual cycle lasting between 25 and 35 days. Exclusion criteria includes any diagnosed chronic disease; use of any medication able to impact energy metabolism or body composition; lactose, gluten and/or soy allergy/intolerance; adherence to a vegetarian, vegan, or restrictive dietary pattern; pregnancy or lactation; use of nutritional supplements in the 2 months prior to study initiation; engagement of more than an hour per day of leisure time physical activity or more than 7 h per week of strenuous activity in the 3 months prior to study initiation; exposure to a nuclear medicine scan or injection of an X-ray dye 1 week prior to study initiation, or a barium test/exam 2 weeks prior to study initiation; and/or diagnosis of claustrophobia.

Potential participants will be instructed to report to the HNRU for a screening visit that includes blood tests (albumin, creatinine, aspartate transaminase, alanine transaminase, sodium, potassium, chloride, and thyroid-stimulating hormone); anthropometric measurements (height, weight, and waist circumference); and the completion of questionnaires eliciting information about health, use of medications, caffeine consumption, physical activity and palatability of the study foods. Only those confident of being able to consume the food items provided during the study period and fulfill the inclusion criteria requirements will be included in the study. Once deemed eligible, participants will be randomly assigned following simple randomization procedure separated by sex to begin with a HP-TDR or CON diet in a 1:1 allocation ratio that will be based on a computer-generated list of random numbers using Microsoft Office Excel 2010 (Microsoft, Redmond, WA, USA). The study team will enroll and assign participants to the interventions.

### Experimental protocol

**Fig. 1:**
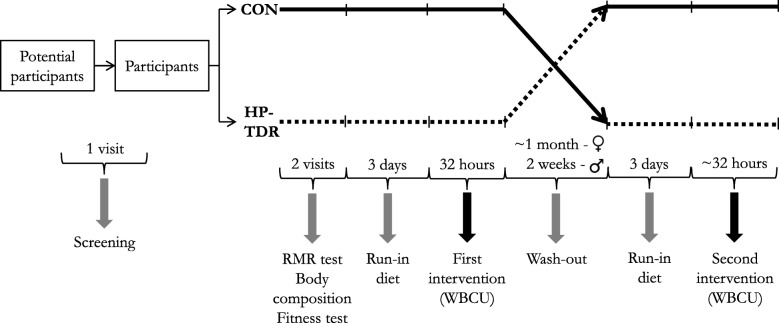
Experimental protocol. Abbreviations: *CON* control diet, *HP-TDR* high-protein total diet replacement, *RMR* resting metabolic rate, *WBCU* whole-body calorimetry unit

**Fig. 2:**
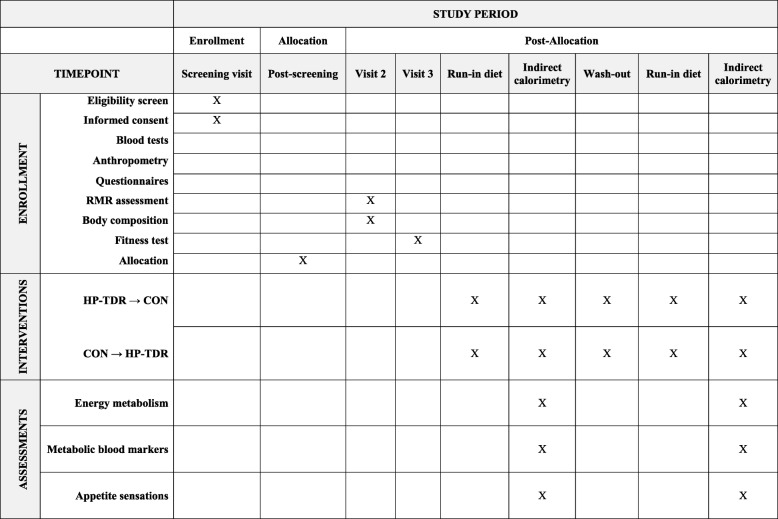
Schedule of enrollment, interventions and assessments (SPIRIT figure). Abbreviation: *CON* control diet, *HP-TDR* high-protein total diet replacement, *RMR* resting metabolic rate

An outline of the experimental protocol is given in Fig. 1, and the schedule of enrollment, interventions, and assessments are provided in Fig. 2. Following the screening process, eligible participants will be invited to attend two study visits for a body composition assessment, a 1-h RMR test, and a fitness test. After these visits are complete, on separate intervention days, participants will undergo two 32-h whole-body calorimetry stays for the measurement of energy metabolism components, appetite sensations, and metabolic blood markers while consuming HP-TDR and CON isocaloric diets in a random order. A 3-day run-in period with a controlled, energy-balanced diet will precede both intervention phases. For women, each intervention phase will be followed by a wash-out period of approximately 1 month to preclude influences of the menstrual cycle. For men, a two-week wash-out period will be required to eliminate any effect of the diet. Participants will be instructed to maintain a stable body weight and physical activity level throughout the study period.

### Anthropometry and body composition

At baseline, the height, weight, waist circumference, and body composition will be assessed. All anthropometric measurements will be performed while the participants are barefoot and wearing light clothing. Height will be measured using a 235 Heightronic Digital Stadiometer (Quick Medical, Issaquah, Wash., USA) to the nearest 0.1 cm. Body weight will be measured using a calibrated digital scale (Health o meter® Professional Remote Display, Sunbeam Products Inc., Fla., USA) to the nearest 0.1 kg. Waist circumference will be measured using a measuring tape.

Body composition will be assessed via dual-energy X-ray absorptiometry (DXA) using a GE Lunar iDXA (General Electric Company, Madison, Wis., USA; enCORE software 13.60 Lunar iDXA GE Health Care®). Participants will be instructed to remove all clothing (except underwear) and metal objects (including underwire bras, piercings, etc.) and put on the hospital gown provided. Whole-body and regional levels of fat mass (FM), lean soft tissue (LST), and bone mineral content (BMC) will be recorded. A previous study (results not published) conducted with this device revealed coefficients of variation (CV) of 1.05% for FM (%), 0.99% for FM (g), 0.37% for LST (g), and 0.40% for BMC (g).

### Fitness test

Prior to the intervention phase, a fitness test will be conducted to personalize and standardize the speed and incline of the prescribed exercise session for each whole-body calorimetry condition. An 8- to 12-h overnight fasting will be required. At the time of the test, participants will be fed a breakfast with the same energy content and macronutrient composition of the CON condition (55% of total energy intake from carbohydrate, 15% from protein and 30% from fat). Breakfast and fitness test starting times will also be identical to the protocol of the whole-body calorimetry conditions (9:00 am and 10:20 am, respectively).

Participants will perform an incremental submaximal exercise test on a treadmill (Freemotion Incline Trainer, Freemotion Fitness, Logan, UT, USA). The fitness test will begin at an individualized walking speed characterized as comfortable and sustainable. After a 5-min warm-up, the incline will be increased by 2% every 3 min, and a constant speed will be maintained until a respiratory exchange ratio (RER) of 0.90 is achieved. During the fitness test, expired gases will be analyzed by a calibrated TrueMax® metabolic measurement system (Parvo Medics TrueOne® 2400 Metabolic Measurement System, Sandy, UT, USA). The heart rate will be monitored continuously with a Polar FT1 Heart Rate Monitor (Polar Electro Oy, Kempele, Finland). To determine the workload for the WBCU exercise session, the RER of the participants will be plotted against their fitness test workload. The speed and incline at which an RER of 0.85 occur will be selected as the intensity for the whole-body calorimetry exercise sessions.

### Run-in diet

A 3-day run-in period with a controlled, energy-balanced diet will precede both 32-h whole-body calorimetry conditions to minimize the effects of any eating style or behavior on baseline data and standardize dietary intake among participants. Diets will be designed by a registered dietitian using the Food Processor Nutrition Analysis Software (version 11.0.124, ESHA Research, Salem, OR, USA). All food will be weighed and prepared at the metabolic kitchen of the HNRU by trained staff. Participants will receive three meals (breakfast, lunch, and dinner) and two snacks (afternoon and evening snacks) per day for 3 days, and they will be instructed to drink water *ad libitum.* They will be required to eat all the food provided, and no additional food will be allowed. The run-in diet will provide 55% of total energy intake from carbohydrate, 15% from protein and 30% from fat, a macronutrient distribution within the Acceptable Macronutrient Distribution Range [3] and similar to a North American dietary pattern [15]. To ensure participants are in energy balance, individual daily energy requirements will be calculated as RMR, determined by a 1-h RMR test using indirect calorimetry, multiplied by a physical activity coefficient, according to the Dietary Reference Intakes [3], and a coefficient of 1.075 representing the metabolizable energy content of the diet [16]. Strenuous physical activity and caffeinated food products will not be allowed during this 3-day run-in period.

### Energy metabolism

Energy expenditure, macronutrient oxidation rates, and balances will be assessed by indirect calorimetry, by measuring the volume of oxygen (VO_2_) and carbon dioxide (VCO_2_), using an open-circuit WBCU. The ambient temperature will be maintained between 21 and 23 °C. Mixed expired air will be drawn out of the unit while fresh conditioned air will be passively drawn into the unit at a predetermined constant flow rate of 60 l per minute. Differences in the VCO_2_ and VO_2_ concentrations of expired and fresh air will be calculated minute-by-minute by the Advance Optima AO2000 Series CO_2_ analyzer (ABB Automation GmbH, Frankfurt, Germany) and the Oxymat 6 O_2_ analyzer (Siemens AG, Munich, Germany). Information will be translated from the gas analyzers to a computer (Acer Aspire AM3910-E3122, Acer Inc., New Taipei City, Taiwan) via the National Instruments NI USB-6221 device (National Instruments Corporation, Austin, Tex., USA) using the PMCSS Software version 1.8 (Pennington Metabolic Chamber Software Suite, Pennington Biomedical Research Center, La., USA). At baseline, participants will complete a 1-h RMR indirect calorimetry test; then, two 32-h tests will be conducted while participants consume the HP-TDR and CON diets.

### RMR test

At baseline, participants will be requested to attend the HNRU after fasting for 8 to 12 h and after refraining from exercise for 24 h prior to the test. Only minimal physical activity will be allowed on the morning of the test (e.g., getting dressed, driving from home to the research unit, and the short walk from the parking lot to the research unit). Participants will be instructed to rest in a supine position, while remaining awake but motionless. RMR will be measured for 60 min with the first 30 min excluded from analysis to account for acclimatization. Results from this test will be used to estimate energy requirements for the 3-day run-in diet and 32-h whole-body calorimetry tests.

### Whole-body calorimetry tests

On the day following the 3-day run-in periods, participants will return to the HNRU after fasting for 8 to 12 h and will spend 32 consecutive hours in the WBCU (8:00 am on day 1 until 4:00 pm on day 2) while receiving the HP-TDR and CON diets in a random order. On the morning of the tests, only minimal physical activity and voiding of the bladder will be requested of the participants before they enter the WBCU. Both 32-h whole-body calorimetry tests will happen during the follicular phase of the women’s menstrual cycles (i.e., between day 6 and 13) and will be conducted approximately 1 month apart (women) and 2 weeks apart (men). The whole-body calorimetry conditions will follow a strict and standard schedule (Additional file 2). Throughout each test, participants will receive three meals (at 9:00 am, 12:00 pm, and 6:00 pm) and two snacks (at 3:00 pm and 9:00 pm). Appetite sensations will be rated immediately before and 30 min after each meal and snack. Blood will be drawn four times (~ 7:30 am, 11:00 am and ~ 2:30 pm on day 1, and ~ 8:00 am on day 2), and urine will be collected for the entire time. On the morning of the first day of the test (10:20 am), a 40-min walking session on a treadmill (BH Fitness T8 SPORT, BH Fitness, Foothill Ranch, Calif., USA) will be completed, at a personalized fixed pace. Sleep will only be allowed during the night (from 10:00 pm to 6:00 am).

### 24-h Energy expenditure and substrate oxidation

 24-h EE and macronutrient oxidation rates will be calculated from the measurements of VO_2_, VCO_2_, and excreted urinary nitrogen (N) by using the formula of Brouwer [17]:

- $$ 24h\  EE\ \left( kcal/ day\right)=3.866\ x\  VO2\ \left(L/ day\right)+1.20\ x\  VCO2\ \left(L/ day\right)-1.43\ x\ N\ \left(g/ day\right) $$
- $$ PROox\ \left(g/ day\right)=6.25\ x\ N\ \left(g/ day\right) $$
- $$ CHOox\ \left(g/ day\right)=4.170\ x\  VCO2\ \left(L/ day\right)-2.965\ x\  VO2\ \left(L/ day\right)-0.390\ x\  PROox\ \left(g/ day\right) $$
- $$ FATox\ \left(g/ day\right)=1.718\ x\  VO2\ \left(L/ day\right)-1.718\ x\  VCO2\ \left(L/ day\right)-0.315\ x\  PROox\ \left(g/ day\right) $$

where PROox, CHOox, and FATox are the protein, carbohydrate, and fat oxidation rates, respectively, in grams per day. Energy and macronutrient balances will be calculated as the difference between intake and oxidation:

- $$ Energy\ Balance= Energy\ Intake\ \left( kcal/ day\right)- Energy\ Expenditure\left( kcal/ day\right) $$
- $$ Macronutrient\ Balance= Intake\ \left(g/ day\right)- Oxidized\ \left(g/ day\right) $$

The respiratory quotient will be calculated as the ratio of VCO_2_ exhaled to VO_2_ inhaled:

- $$ RQ=\frac{VCO2\ (L)}{VO2\ (L)} $$

During each whole-body calorimetry test, the following components of participants’ energy expenditure will be assessed: 24-h EE, RMR, BMR (energy expended to maintain essential vital function), SMR, postprandial EE, and exercise EE. The RMR will be assessed as described above. The BMR will be assessed in the morning of the second day of the test. Participants will receive a wake-up call at 6:00 am and will be instructed not to get up from bed but to rest in a supine position while awake but motionless for 60 min. The SMR will be computed for a 3-h period (2:00 am to 5:00 am), and the sleep EE will be extrapolated to an 8-h sleep night. Postprandial EE will be assessed on the second day of the test for the 6 consecutive h after the participants have finished their breakfasts. Exercise EE will be assessed in the morning of the first day of the test for 40 min during the exercise session (10:20 am to 11:00 am).

### Experimental diets

The HP-TDR and CON diets that will be consumed during both 32-h whole-body calorimetry conditions will be designed by a registered dietitian using the same approach as per the run-in diet. All food will be weighed and prepared by trained staff. Participants will receive three meals (breakfast, lunch, and dinner) and two snacks (afternoon and evening snacks) throughout each whole-body calorimetry test (Table 1). Bottled water will be provided *ad libitum.* The CON diet will provide 55% of total energy intake from carbohydrate, 15% from protein, and 30% from fat, and the HP-TDR diet will provide 35% of total energy intake from carbohydrate, 40% from protein and 25% from fat. Table 2 illustrates the nutrient composition of a typical 2000 kcal diet. The CON diet will be comprised of food items that can be found at the local grocery stores and the HP-TDR diet will consist of a soy-protein nutritional supplement (Almased®, Almased USA, Inc., St. Petersburg, FL, USA) mixed with olive oil and low-fat milk (1% fat) for the main meals and with olive oil and apple juice for the snacks, per label instructions [18]. The first dietary intervention randomly offered in the WBCU will be designed to maintain participants in energy balance and the energy content of each meal and snack will be identical for the HP-TDR and CON diets (eucaloric). Energy intake will be initially estimated using a 1-h RMR test, and it will be adjusted throughout the day on the basis of measured EE using WBCU data points. When the prescribed energy intake falls outside ±100 kcal of the predicted 24-h EE, the amount of calories provided in the dinner and evening snack will be adjusted (added or subtracted) in an attempt to achieve energy balance. Participants will be required to consume all the food provided and meal trays will be checked after consumption.

**Table 1:** Composition of the CON and HP-TDR diets

Meals	CON Diet	HP-TDR
Breakfast	Whole wheat bread	HP-TDR powder
	Peanut butter	Low-fat (1%) milk
	Orange juice	Olive oil
	Low-fat (1%) milk^1^	
	Boiled egg^1^	
Lunch	Turkey wrap	HP-TDR powder
	*Flour tortilla*	Low-fat (1%) milk
	*Deli style turkey*	Olive oil
	*Cheese*	
	*Lettuce*	
	*Tomato*	
	*Olive oil*	
	Tomato soup	
	Canned peaches^1^	
	Yogurt^1^	
Afternoon snack	Apple	HP-TDR powder
	Crackers	Apple juice
	Cheese	Olive oil
	Yogurt^1^	
Dinner	Chicken stir fry	HP-TDR powder
	*Chicken breast*	Low-fat (1%) milk
	*Celery*	Olive oil
	*Carrot*	
	*Onion*	
	Brown rice	
	Yogurt^1^	
Evening snack	Cereal	HP-TDR powder
	Low-fat (1%) milk	Apple juice
	Almonds	Olive oil
	Canned peaches^1^	

^1^Food items added or not, depending on the energy requirements of the participants

Abbreviations: *CON* control, *HP-TDR* High-protein total diet replacement

**Table 2:** Nutrient composition of a typical study diet

	HP-TDR	CON Diet
Energy		
*kcal/day*	2000	2000
Protein		
*g/day*	200	76
*% of TEI*	40	15
Fat		
*g/day*	55	67
*% of TEI*	25	30
Carbohydrate		
*g/day*	175	280
*% of TEI*	35	55

Abbreviations: *BW* body weight, *CON* control, *HP-TDR* high-protein total diet replacement, *TEI* total energy intake

### Appetite sensations

During each whole-body calorimetry condition, participants will rate their appetite sensations (hunger, satiety, fullness, and prospective food consumption) using a validated anchored 100-mm visual analog scale (VAS) [19]. Questionnaires will be administered using the paper-and-pen method, and participants will be instructed to make a single vertical mark at the appropriate point between the two anchors on each scale to indicate the intensity of their subjective states regarding each element. Participants will be asked to indicate, on a scale from 0 to 100 mm, how they feel at the moment they complete these questions: How hungry do you feel? (I am not hungry at all – I have never been more hungry); How satisfied do you feel? (I am completely empty – I cannot eat another bite); How full do you feel? (not at all full – totally full); How much do you think you can eat? (nothing at all – a lot). Participants will complete the questionnaires immediately before and 30 min after each meal (breakfast, lunch, and dinner) and snack (afternoon and evening). For each rating period, a new questionnaire will be provided to participants, and they will not be allowed to consult their previous ratings.

### Blood and urine analysis

Blood will be sampled by venipuncture from participants at four time points during each whole-body calorimetry condition: 1) the morning on the first day of the test (Baseline); 2) immediately after the exercise session (4 h post-baseline); 3) two hours after lunch (7.5 h post-baseline); and 4) the morning on the second day of test (24 h post-baseline). Both morning blood draws will be sampled from subjects after a 10-h overnight fast. Blood will be drawn into BD Vacutainer® blood collection tubes (Becton, Dickinson and Company, Franklin Lakes, NJ, USA), spray-coated with silica and a polymer gel for serum separation or with K_2_-ethylenediaminetetraacetic acid (EDTA) for plasma separation. Before centrifugation, a protease inhibitor 4-(2-aminoethyl)benzenesulfonyl fluoride hydrochloride (AEBSF) (Sigma-Aldrich, Oakville, ON, Canada) will be added to the K_2-_EDTA tubes. Samples will be centrifuged at 450 xg for 10 min and aliquoted for storage at −80 °C for the subsequent measurement of biomarkers. Hydrochloric acid (1 N, 100 μL) will be added to the ghrelin aliquot prior to storage. Serum samples will be analyzed for glucose, insulin, cholesterol, HDL cholesterol, LDL cholesterol, triglyceride, and non-HDL cholesterol by DynaLIFE Medical Labs (Edmonton, AB, Canada). Plasma samples will be analyzed for ghrelin, PYY, GLP-1, leptin, free glycerol and non-esterified fatty acids (NEFA) at the HNRU (University of Alberta, Edmonton, AB, Canada). Leptin and GLP-1 (active) will be measured by electrochemiluminescence using the MULTI-ARRAY® Assay System (Meso Scale Discovery®, Gaithersburg, Md., USA) and V-PLEX® (Meso Scale Discovery®, Gaithersburg, Md., USA), respectively. Ghrelin (active) and PYY will be measured using enzyme-linked immunosorbent assay (ELISA) kits from EMD Millipore Co. (Billerica, Mass., USA). Free glycerol and NEFA will be measured using enzyme immunoassay kits from Zen-Bio Inc. (Research Triangle Park, N.C., USA).

Urine will be collected for the entire time from the participants while at the WBCU for the measurement of urinary N. Collections will be initiated after the first morning urinary void and collected into sterile urine jugs. During collection, urine will be stored at −1 °C inside a refrigerator located in the WBCU and will be taken to the laboratory after each whole-body calorimetry test ends. Urine volume will be measured, and aliquots (~ 1.5 mL each) will be placed into tubes for storage at −80 °C before analysis. Total urinary N will be determined by chemiluminescence using a high temperature Shimadzu TOC-L CPH Model Total Organic Carbon Analyzer with an ASI-L autosampler and TNM-L unit (Shimadzu Corporation, October 2015. Suzhuo, Jiangsu, China).

### Statistical analysis

#### Sample size estimate

The sample size estimation was conducted separately for each sex. A total of 12 participants will enable detection of an effect size of 1.41, which was calculated based on differences in RQ between dependent groups receiving a HP-TDR (0.85 ± 0.03) or maintaining usual dietary intake (0.90 ± 0.04) from a previously published study [20]. A two-sided t-test achieves 88% power to infer that the mean difference is not 0.05 when the total sample size of a 2 × 2 crossover design is 12, the actual mean difference is 0.06, the standard deviation of the paired difference is 0.01, and the significance level is 0.05. Accounting for a 20% attrition rate, a total sample size of 14 participants of each sex would be needed to complete the study. An alternative sample size calculation was conducted based on differences in fat balance between dependent groups receiving a HP-TDR (5.55 ± 4.20 g/day) or maintaining usual dietary intake (10.25 ± 4.19 g/day) using unpublished data from Koohkan et al. (2012) [20]. Group sample sizes of 19 in each arm achieves 90% power to detect a difference of -4.7 g/day with a significance level of 0.05 using a two-sided Mann-Whitney test. Assuming a 20% attrition rate, a total 23 participants would be needed in each group. The sample size calculation was done using PASS Power Analysis and Sample Size software version 19.0.1 (NCSS Statistical Software, Kaysville, Utah, USA).

#### Data analysis

IBM® SPSS® Statistics version 24 (International Business Machines Corporation, New York, NJ, USA) will be used to perform statistical analyses. Differences will be regarded as statistically significant if *p* < 0.05. Missing data will be omitted, and the remaining data will be analyzed. To determine whether a statistically significant mean difference exists between the HP-TDR and CON groups, a paired-samples t-test or Wilcoxon signed rank test will be used, depending on the data distribution. Shapiro Wilk’s test will be used to check the normality of the data. Inspection of a boxplot for values greater than 1.5 box-lengths from the edge of the box will be done to detect outliers. A two-way repeated measures analysis of variance (ANOVA) will be carried out to determine the effect of the dietary interventions on appetite sensations. Outliers will be detected by studentized residuals greater than ±3 standard deviations. Sphericity will be assessed for the interaction term by the Mauchly’s test of sphericity. The relationships between variables will be determined by a Pearson’s product-moment correlation. To determine whether the order of treatment has an effect on the results, a repeated measures ANOVA with the order of treatment as the between-subjects factor will be performed.

### Data monitoring

This will be an acute dietary intervention study, and any adverse event and/or any unintended effect of the trial intervention or trial conduct is unlikely to happen. Possibly, some participants will feel some discomfort in their muscles after walking on the treadmill; however, the activity is moderate. Blood draws can cause injury and a small risk of infection, which are minimized with proper procedures. Participants may also feel uncomfortable being alone in the WBCU; however, research staff will always be nearby, and an intercom system is available. The X-ray dose associated with DXA scan is very low and safe.

If abnormal results from the questionnaires or blood tests are noticed, the study team will follow the University Emergency procedures, as required. If a participant requires a medical referral, the physician involved in this clinical trial will assess the situation and refer the participant as required to the appropriate health care professional. If participants become ill or injured as a result of being in this study, they will receive any necessary medical treatment.

### Dissemination policy

The results of this study will be disseminated to researchers, health professionals and the general public by publication in peer-reviewed national and international journals, as well as by poster and oral presentations at conferences and meetings on nutrition and health. Authorship will follow the recommendations of the International Committee of Journal Editors.

## Discussion

Obesity is an important healthcare problem caused by an imbalance between calories consumed and expended over time [21, 22]. To date, treatment strategies for obesity are lacking. Protein intake above the currently recommended amount [3] may confer a metabolic advantage by increasing energy expenditure and satiety; however, a lack of dietary adherence to a HP diet seems to affect its long-term effectiveness [6]. TDR have been indicated as long-term therapeutic strategies to treat overweight and obesity [8, 9]. Considering these strategies in combination, a HP TDR seems to be an interesting approach to treat overweight and obesity, promoting effective weight loss and its maintenance over the years. As mentioned previously, this research is novel. When compared to individuals receiving a conventional diet with a typical North American macronutrient distribution with the same energy content, those receiving a HP diet comprised of a TDR are hypothesized to be in negative fat balance, have increased 24-h EE, improved metabolic profiles, and feel more satiated.

Investigating the impact of a HP diet comprised of a TDR in healthy individuals of both sexes will allow a better understanding of the effects of this strategy in a normal physiological condition. These data can then be used as reference for studies testing the effects of HP TDR in overweight and obese individuals with and without comorbidities. Therefore, the results of this clinical trial can ultimately be used to develop future strategies aimed at optimizing diet quality and weight management. Strategies aimed at the prevention of overweight, obesity, and related chronic diseases are important for the improvement of health and quality of life, also ultimately addressing the growing financial burden of obesity and related diseases on the health care system.

## Trial status

Enrollment of women started in November 2016 and was concluded in March 2018. Enrollment of men started in August 2018 and is expected to finish in December 2019. The protocol versions are 1) NCT02811276: version 10 (2 March 2018) and 2) NCT03565510: version 3 (28 September 2018).

## Supplementary information

**Additional file 1.** SPIRIT 2013 Checklist: Recommended items to address in a clinical trial protocol and related documents*.

**Additional file 2.** Schedule of the indirect calorimetry tests.

## Data Availability

Data sharing is not applicable to this article because no datasets were generated or analyzed during the current study.

## Abbreviations

AEBSF: 4-(2-aminoethyl)benzenesulfonyl fluoride hydrochloride
ANOVA: analysis of variance
BMC: bone mineral content
BMI: body mass index
BMR: basal metabolic rate
CHOox: carbohydrate oxidation
CON: control
CV: coefficient of variation
DXA: dual-energy X-ray absorptiometry
EDTA: ethylenediaminetetraacetic acid
EE: energy expenditure
ELISA: enzyme-linked immunosorbent assay
FATox: fat oxidation
FM: fat mass
GLP-1: glucagon-like peptide-1
HDL: high-density lipoprotein
HNRU: Human Nutrition Research Unit
HP: high-protein
LDL: low-density lipoprotein
LST: lean soft tissue
N: nitrogen
NEFA: non-esterified fatty acids
PROox: protein oxidation
PYY: peptide tyrosine-tyrosine
RER: respiratory exchange ratio
RMR: resting metabolic rate
RQ: respiratory quotient
SMR: sleeping metabolic rate
SPIRIT: Standard Protocol Items: Recommendations for Interventional Trials
TDR: total diet replacement
VAS: visual analog scale
VCO_2_: volume of carbon dioxide
VO_2_: volume of oxygen
WBCU: whole body calorimetry unit
