# Supplementary material for: Examining the effects of a high-protein total diet replacement on energy metabolism, metabolic blood markers, and appetite sensations in healthy adults: protocol for two complementary, randomized, controlled, crossover trials
Source: Trials. 2019 Dec 27;20:787. doi: 10.1186/s13063-019-3950-y (PMC6935127; doi:10.1186/s13063-019-3950-y)
Supplement: Supplementary file 2 — Additional file 2. Schedule of the indirect calorimetry tests. [file 13063_2019_3950_MOESM2_ESM.docx]

**Additional File 2.** Schedule of the indirect calorimetry tests

| **TIME** | **ACTIVITIES** |
| --- | --- |
| **DAY 1** | |
| 8:00 a.m. | Test begins - 60 minutes of rest on bed |
| 9:00 a.m. | Appetite sensation assessment |
| 9:00 – 9:30 a.m. | Breakfast |
| 9:30 a.m. | Appetite sensation assessment |
| 9:30 – 10:20 a.m. | Leisure/work time (TV, computer, reading) |
| 10:20 – 11:00 a.m. | Exercise session on treadmill |
| 11:00 a.m. | Blood draw |
| 11:00 – 12:00 p.m. | Leisure/work time (TV, computer, reading) |
| 12:00 p.m. | Appetite sensation assessment |
| 12:00 – 12:30 p.m. | Lunch |
| 12:30 p.m. | Appetite sensation assessment |
| 12:30 – 2:30 p.m. | Leisure/work time (TV, computer, reading) |
| 2:30 p.m. | Blood draw |
| 2:30 – 3:00 p.m. | Leisure/work time (TV, computer, reading) |
| 3:00 p.m. | Appetite sensation assessment |
| 3:00 – 3:30 p.m. | Afternoon snack |
| 3:30 p.m. | Appetite sensation assessment |
| 3:30 – 6:00 p.m. | Leisure/work time (TV, computer, reading) |
| 6:00 p.m. | Appetite sensation assessment |
| 6:00 - 6:30 p.m. | Dinner |
| 6:30 p.m. | Appetite sensation assessment |
| 6:30 – 9:00 p.m. | Leisure/work time (TV, computer, reading) |
| 9:00 p.m. | Appetite sensation assessment |
| 9:00 – 9:30 p.m. | Evening snack |
| 9:30 p.m. | Appetite sensation assessment |
| 9:45 p.m. | Get ready for bed |
| 10:00 p.m. – 6:00 a.m. | Sleep |
| **DAY 2** | |
| 6:00 am | Wake up call |
| 6:00 – 7:00 a.m. | 60 minutes of rest on bed |
| 7:00 – 7:25 a.m. | Participant can use the washroom, brush teeth, change clothes, etc. |
| 7:30 – 8:30 a.m. | 60 minutes of rest on bed |
| 8:30 – 8:35 a.m. | Blood draw |
| 8:35 a.m. | Appetite sensation assessment |
| 8:40 – 9:00 a.m. | Breakfast |
| 9:30 a.m. | Appetite sensation assessment |
| 9:00 – 3:00 p.m. | Participants seated or lying down without too much movement |
| 3:00 p.m. | Participant get ready to leave the WBCU |
| 3:10 p.m. | Participant leaves the WBCU |

Abbreviations: WBCU: whole-body calorimetry unit.
